# Bilateral Wallerian Degeneration of the Pontocerebellar Tracts

**DOI:** 10.1155/2015/970570

**Published:** 2015-06-10

**Authors:** Azad Hekimoglu, Ihsaniye Suer Dogan, Aynur Turan, Mehmet Fevzi Oztekin, Baki Hekimoglu

**Affiliations:** ^1^Department of Radiology, Diskapi Yildirim Beyazit Training and Research Hospital, Ankara, Turkey; ^2^Department of Neurology, Diskapi Yildirim Beyazit Training and Research Hospital, Ankara, Turkey

## Abstract

Wallerian degeneration is the process of progressive demyelination and disintegration of the distal axonal segment following the transection of the axon or damage to the neuron. We report a case of a patient with Wallerian degeneration of the pontocerebellar tracts. She had a history of a pontine infarction 3 months ago. Wallerian degeneration of pontocerebellar tracts is seen bilaterally and symmetrically and is more visible in the middle cerebellar peduncles. Along the middle cerebellar peduncles hyperintense signal was detected on T2 weighted images. Wallerian degeneration of pontocerebellar tracts is a rare entity. It can occur bilaterally after a large pontine infarction. Magnetic resonance imaging seems to be the most effective technique for detection of Wallerian degeneration. In this report we want to mention this rare entity and to prevent wrong diagnosis.

## 1. Introduction

Wallerian degeneration represents a uniform answer to injury within the central and peripheral nervous systems. Infarction is the most common event resulting in WD in the central nervous system. Magnetic resonance imaging seems to be the most effective technique for detection of Wallerian degeneration. Along the affected white matter tractus high signal was detected on both T2 weighted images and fluid-attenuated inversion recovery images.

## 2. Case Presentation

A 73-year-old woman was admitted to emergency service with headache, vertigo, speech difficulties, and right-sided weakness. Patient had no history of arterial hypertension, hypercholesterolemia, and diabetes mellitus.

A transthoracic echocardiogram was performed and findings were normal for her age.

**Figure 1:**
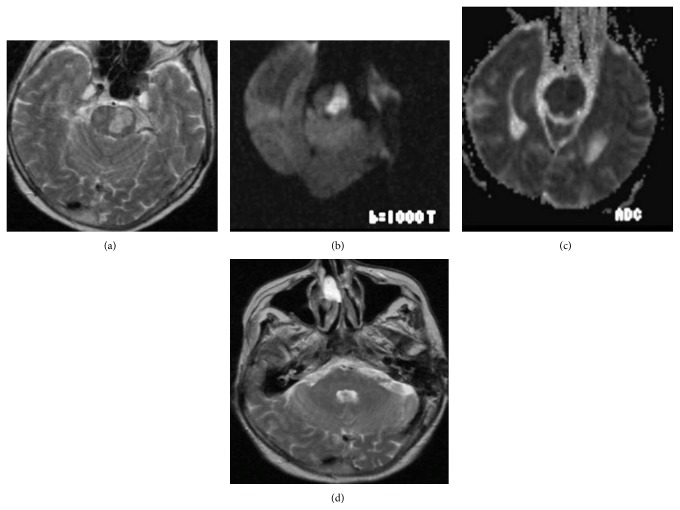
Left paramedian acute pontine infarction: axial T2WI (a) hyperintense signal on the left side of the pons, axial DWI (b) and ADC maps (c) restricted diffusion, and axial T2WI (d) MCPs were normal.

A brain MRI was performed and demonstrated a left, paramedian pontine acute infarct (Figure 1). On T2WI hyperintensities were seen on the left side of the pons (Figure 1(a)). On DWI and ADC maps restricted diffusion (Figures 1(b)-1(c)) was seen which showed the acute infarct.

After 3 months the patient was admitted to the emergency service again with worsening of her symptoms. Speech difficulties, dysarthria, and right hemiparesis on both upper and lower limbs were depicted.

**Figure 2:**
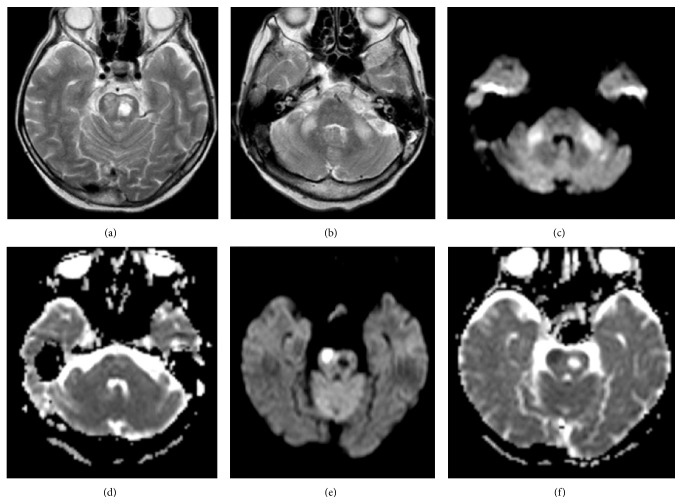
3 months after left paramedian acute pontine infarction. Axial T2WI (a) hyperintense signal in pons due to chronic infarct. Axial T2WI (b) bilateral and symmetrical hyperintense signal in MCPs due to WD. Axial DWI (c) and ADC map (d) shows hyperintense signal. Axial DWI (e) and ADC map (f) shows restricted diffusion due to new acute infarct in the right side of the pons.

A second brain MRI was performed (Figure 2). On MRI a left sided pons encephalomalacia was seen secondary to pontine infarction (Figure 2(a)). At the same time a new focus of acute infarct was seen on the anterior right side of the pons (Figures 2(e)-2(f)). On T2WI and FLAIR images bilateral and symmetrical hyperintensities along the middle cerebellar peduncles were seen. These lesions were interpreted as WD of pontocerebellar tracts due to previous pontine infarction (Figure 2(b)).

Along the MCPs lesions were hyperintense on both DWI and ADC maps, consistent with T2 shine-through effect (Figures 2(c)-2(d)).

## 3. Discussion

WD represents a uniform answer to injury within the central and peripheral nervous system. In the first days following the injury disintegration of axonal structures occurs, after several weeks infiltration of macrophages and degradation of myelin formed, and finally fibrosis and atrophy of the affected fiber tracts appear [1].

WD is most frequently observed in the corticospinal tract following infarction of the motor cortex or internal capsule [2].

Infarction is the most common event resulting in WD in the central nervous system. Also hemorrhage, necrosis, neoplasm, trauma, focal demyelination, white matter disorders, and multisystem atrophy are reported entities that may cause degeneration [3].

After infarction it usually takes two to four weeks before WD can be detected by conventional MRI [1]. Conventional MRI and DWI depict WD when sufficiently large bundles of fibers are involved along the corticospinal tract, the corpus callosum, fibers of the optic radiations, fornices, and cerebellar peduncles [4, 5].

The main finding is a hyperintensity on T2WI and FLAIR images along the affected tracts.

WD of cerebellar peduncles is rarely described. It usually involves the middle ones because they are largest and the main path for pontocerebellar tracts [6]. The MCPs are a massive bundle of fibers connecting the basal portion of the pons with the cerebellum.

WD of the unilateral pontine infarction results in bilateral symmetrical hyperintense lesions secondary to the crossed distribution of the pontocerebellar fibers [7].

Only few reports have described the MR findings of WD in the middle cerebellar peduncles following pontine infarction, hemorrhage, and acute vascular lesions [7, 8].

In our case after a unilateral pontine infarct in the MCPs on T2WI bilateral and symmetrical hyperintense lesions were seen owing to WD.

Bilateral symmetric lesions of the MCPs can also be seen in other conditions, such as central pontine myelinolysis, neurodegenerative disorders, multisystem atrophy, and progressive multifocal leukoencephalopathy. However, clinic and biochemical investigations help in differentiation.

In conclusion the basic understanding required to diagnose WD in the brain is a detailed knowledge of the course of the association fibers.

High signal in the MCPs following a pontine lesion is almost certainly attributable to WD of the pontocerebellar tracts and should not be mistaken for a new infarction.

WD is a secondary lesion and should not be mistaken for a primary, independent lesion.

## Abbreviations

WD: Wallerian degeneration
MRI: Magnetic resonance imaging
MCPs: Middle cerebellar peduncles
T2WI: T2 weighted images
DWI: Diffusion weighted images
ADC: Apparent diffusion coefficient
FLAIR: Fluid-attenuated inversion recovery.
